# Liver surgery in the presence of cirrhosis or steatosis: Is morbidity increased?

**DOI:** 10.1186/1754-9493-2-8

**Published:** 2008-04-25

**Authors:** Lucas McCormack, Pablo Capitanich, Emilio Quiñonez

**Affiliations:** 1 Hepato-Pancreato-Biliary and Liver Transplantation Unit, General Surgery Service, Hospital Aleman, Av. Pueyrredón 1640 (1118), Ciudad Autónoma de Buenos Aires, Argentina

## Abstract

**Background data:**

The prevalence of steatosis and hepatitis-related liver cirrhosis is dramatically increasing together worldwide. Cirrhosis and, more recently, steatosis are recognized as a clinically important feature that influences patient morbidity and mortality after hepatic resection when compared with patients with healthy liver.

**Objective:**

To review present knowledge regarding how the presence of cirrhosis or steatosis can influence postoperative outcome after liver resection.

**Methods:**

A critical review of the English literature was performed to provide data concerning postoperative outcome of patients presenting injured livers who required hepatectomy.

**Results:**

In clinical studies, the presence of steatosis impaired postoperative outcome regardless the severity and quality of the hepatic fat. A great improvement in postoperative outcome has been achieved using modern and multidisciplinary preoperative workup in cirrhotic patients. Due to the lack of a proper classification for morbidity and a clear definition of hepatic failure in the literature, the comparison between different studies is very limited. Although, many surgical strategies have been developed to protect injured liver surgery, no one have gained worldwide acceptance.

**Conclusion:**

Surgeons should take the presence of underlying injured livers into account when planning the extent and type of hepatic surgery. Preoperative and perioperative interventions should be considered to minimize the additional damage. Further randomized trials should focus on the evaluation of novel preoperative strategies to minimize risk in these patients. Each referral liver center should have the commitment to report all deaths related to postoperative hepatic failure and to use a common classification system for postoperative complications.

## 

Liver surgery is the only curative treatment of patients with liver tumors [1-3]. Developments in surgical techniques and advances in the management of critical patients have increased the number of potential candidates for surgery [4]. High-volume centers have convincingly reported a dramatic decrease in perioperative mortality after liver resection over the past two decades [5,6]. Even zero mortality can be achieved with systematic preoperative patient selection [1].

As a result of better postoperative outcome, indications for liver surgery and the extent of liver resection have expanded and currently major liver resections are routinely performed despite the presence of underlying liver disorders [6]. While major hepatic resection (≥ 3 liver segments) can be safely performed on healthy livers, the risk of such resection in patients with underlying hepatic disease remains unclear. The available literature shows a great dispersion in postoperative mortality rates associated with liver failure after hepatic surgery in either sick or healthy liver [7,8]. Moreover, the lack of a clear definition of postoperative liver failure, the use of different classification systems to assess postoperative complications and the absence of reliable parameters to estimate the remnant liver volume necessary to avoid hepatic failure makes the interpretation of the literature a difficult challenge [7,9,10].

A potentially increased risk of impaired postoperative recovery has been suggested for patient with cirrhosis [11] and recently, also for those with liver steatosis [12]. The objective of this review is to present the knowledge regarding how the presence of cirrhosis or steatosis can influence postoperative outcome after liver resection.

## Steatotic livers

By definition hepatic steatosis is characterized by an accumulation of lipids in the liver and is related to a spectrum of etiologic features such as obesity, diabetes, excessive use of alcohol, and a variety of drugs and toxins [13,14]. Fatty accumulation is considered pathologic when the hepatic fat content, consisting mainly of triglycerides, exceeds 5% of the actual wet weight of liver [15].

In a retrospective study of a large series of hepatic resections covering a decade (1991–2001), liver steatosis was found to be the most common underlying hepatic abnormality [6]. Even though the global prevalence has yet to be evaluated, studies reported prevalence of 10% to 20% in lean population, 60% to 74% among the obese (i.e. body-mass index ≥ 30 kg/m^2^) and over 90% in the morbidly obese (i.e. body-mass index ≥ 40 kg/m^2^) [16-20]. Approximately, 3% of lean children are affected and the prevalence increases up to 53% among obese children [13,21]. Incidence of steatohepatitis ranges from 3% in lean population, to 18% among obese to almost 50% in morbidly obese individuals. In patients with steatohepatitis, the risk to develop cirrhosis is 10% to 30% and is associated with a decreased 5- and 10-year survival of 67% and 59%, respectively [15]. Moreover, the prevalence of steatosis and steatohepatitis is expected to dramatically increase in the near future due to increasing obesity among the Western population.

### Diagnosis

Steatosis is usually an incidental finding and hepatomegaly is often the only finding on physical examination. Despite widespread clinical use of imaging methods, ultrasound, computed tomography, or magnetic resonance imagining can only to some extent detect the degree of steatosis. Saadeh et al demonstrated the limitation of radiological modalities in a study where only steatosis >25–30% could be reliably detected radiologically [22]. In addition, none of these modalities was able to either distinguish the grading or the type of liver fat or to detect the individual pathologic features important to establish steatohepatitis such as necro-inflammatory changes, hepatocyte ballooning, and fibrosis [23].

There is consensus that the gold standard of diagnosis is = 2 liver biopsies, as a single biopsy can result in substantial misdiagnosis and staging inaccuracies [24]. However, due to the risk of fatal bleeding after liver biopsy (0.4%), it is not routinely performed in patients without apparent complicated liver disease [25]. A uniform quantitative grading for steatosis and steatohepatitis has been suggested, combining the identified key pathologic features [26]. Besides quantitative grading, steatosis can be classified qualitatively into microvesicular and macrovesicular forms. Most frequent is the macrovesicular one, and is frequently associated with obesity, non-insulin-dependent diabetes, some dyslipidemias, and alcohol abuse. Microvesicular steatosis is usually related to more acute conditions such as acute viral infections, metabolic disorders, and various toxins but also to acute fatty liver of pregnancy. The histopathologic features of steatosis are evaluated in preoperative needle biopsies or operative wedge specimens that are frozen and/or deparaffinized. The staining methods currently used are hematoxylin and eosin with which the fatty changes are assessed by considering the non-stained regions. In addition, specific fat stains such as Oil Red O and Sudan III are used. However, there are several problems in clinical application of these staining methods. The conventional techniques potentially underestimate the extent of fatty infiltration as they fail to identify microvesicular forms of steatosis [27]. Also the fat specific stains have pitfalls, for example, in Oil Red O-stained liver tissue, the quality and quantity of the staining are highly operator-dependent and false-positive results or overestimation of the severity are possible because of unspecific sinusoidal staining [28].

### Associated Clinical Conditions

Patients with nonalcoholic fatty liver disease have an increased prevalence of non-insulin-dependent diabetes, but the actual role of diabetes in postoperative recovery is unclear as studies report contradictory results. Non-insulin-dependent diabetes was identified as independent and significant variable predicting major postoperative complications in a cohort of 209 patients undergoing liver resection [29]. However, contrary to this study, a study including 525 diabetic and non-diabetic patients requiring hepatectomy, reported no difference in perioperative morbidity or mortality and no effect was observed in long-term prognosis [30]. Although the impact of diabetes on postoperative complications remains unclear, an increased rate of wound infections was reported in an impressive cohort of over 20,000 patients [31]. From the analysis of a large database, was concluded that a subset of diabetic patients having steatosis in the analysis of the liver specimen, poorly tolerate major liver resection [32]. Thus, when contemplating major hepatectomy in patients with diabetes mellitus, surgeons should be aware that the presence of diabetes mellitus may lead to higher incidence of hepatic failure postoperatively.

Obesity is crucially linked with steatosis [14]. In the past, obesity has been also linked to increased perioperative technical complications leading to prolonged postoperative recovery. However, Dindo et al prospectively investigated a cohort of 6336 patients undergoing elective major surgery and found no increase in postoperative morbidity and mortality between obese and non-obese, not even in morbidly obese patients [33]. However, the authors did not discriminate patients who underwent exclusively liver surgery. Unfortunately, reports referred to perioperative outcome of obese patients requiring hepatectomy are scarce in the literature.

In contrast to general obesity, body fat accumulation (subcutaneous or intraabdominal) has been reported to be independently associated with postoperative morbidity in a prospective study of 139 patients who underwent gastric or colorectal cancer surgery [34].

### Post-Operative Complications

Only a few studies have focused on liver steatosis as a risk factor for postoperative complications after liver resection (Table 1). However, there are some general problems in the reporting of these studies. The histopathology methods for diagnosis of steatosis are not frequently, if ever, mentioned. So, the reliability of diagnosis of steatosis remains uncertain, rendering the comparison of the results difficult. Uniform grading together with a more detailed description of the staining methods used and the number and sort of biopsies taken are important to compare different studies [15]. In the absence of a uniform classification system accepted by most to better stratify morbidity by severity, scarce data is reported concerning the incidence of major complication rate after liver resection in steatotic patients. Finally, mortality is usually assessed as in-hospital mortality or 60 days mortality after operation, and there are no studies evaluating 5- or 10-year survival of steatotic patients after surgey.

**Table 1:** Reported studies focus on postoperative outcome after liver resection of patients with steatotic livers.

Authors	Year	Center	Grading of steatosis (%)	Number of patients	Overall Complication (%)	*P *value	Mortality (%)	*P *value
Behrns et al (35)	1998	Mayo Clinic. Rochester, USA.	None	72	10	NA	3	NA
			Mild (<30)	56	14		7	
			Marked (≥ 30)	7	29		14	
Belghiti et al (5)	2000	Hopital Beaujon. Paris, France.	None	441	8	0.003	1	0.5
			Steatosis (≥ 30)	37	22		0	
Jamargin et al (6)	2002	MSKCC. New York, USA.	None	1275	48	0.197	3.9	0.309
			Steatosis (≥ 30)	380	44		2.8	
Kooby et al * (36)	2003	MSKCC. New York, USA.	None	160	35	<0.01	3.1	0.29
			Mild (<30)	223	48		3.6	
			Marked (≥ 30)	102	62		5.9	
Mc Cormack et al * (12)	2007	Swiss HPB Center. Zurich, Switzerland.	None	58 ^@^	6.9#	0.001	1.7	0.21
			Steatosis (≥ 10)	58 ^@^	27#		8.6	

NA: Not Available; * Case matched analysis; # Referred only to major complication rate using Dindo et al classification (10); @ Only major liver resection were analyzed (≥ 3 liver segments)

In 1998, Behrns et al evaluated in a retrospective study of 135 patients, the safety of major resection in patients with hepatic steatosis [35]. They reported an increased postoperative mortality, morbidity, and blood transfusion rates together with longer operative time in the presence of steatosis of ≥ 30%. Belghiti et al, in a large database including 478 liver resections, detected the presence of hepatic steatosis ≥ 30% in 37 (7.7%) patients, and they demonstrated that steatosis was an independent risk factor for postoperative complications but not for mortality [5]. In the analysis of a cohort of 1803 liver resections, the histological analysis demonstrated that only 55% of the liver specimens had normal parenchyma and 18% had liver steatosis [6]. In contrast, the presence of steatosis was not a risk factor for postoperative outcome after hepatectomy. Later, Kooby et al reviewed the above-mentioned cohort to perform a case-matched study involving 325 steatotic patients compared with 160 with normal liver who were matched according to age, co-morbidities, and the extent of resection [36]. Interestingly, they showed that steatosis was a predictor of postoperative complications, but mainly due to minor complications. Major postoperative complications, eg, requiring major therapeutic intervention or mortality, were not affected by the presence of steatosis. To note, information regarding the presence of concomitant risk factors, such as nutritional parameters, kidney function and cholestasis, were not included in this analysis. Furthermore, the histologic analysis did not differentiate between the two types of liver steatosis or other histological features such as steatohepatitis or fibrosis. Therefore, we decided to investigate the influence of the quantitative and qualitative analysis of steatosis in postoperative outcome after major liver resection [12]. In our study, each patient with liver steatosis was matched with a similar patient with normal liver who required major hepatectomy according to the following variables: age, gender, ASA (American Society of Anesthesiologists) score, diagnosis, extent of hepatectomy, and the need of bilio-enteric bypass. We found that steatosis was the most significant preoperative risk factor for postoperative complications and should be considered for the planning of a major liver resection. Interestingly, neither the type nor the grading of liver steatosis influenced postoperative outcome after major hepatectomy. A novel finding in this study was that the presence of preoperative jaundice in a steatotic liver was an independent risk factor for mortality after surgery. Consequently, efforts should be made either to postpone surgery until normalization of bilirubin or to minimize the extent of liver resection in those patients (e.g., economic hepatectomy combined with ablation therapies, two-stage hepatectomy, etc).

## Cirrhotic livers

Reduction of postoperative morbidity and mortality is still a great challenge in cirrhotic patients. Today, an improvement in perioperative outcome of cirrhotic patients undergoing liver surgery has been achieved as a result of a continuous advance in preoperative imagining, surgical technique, anesthesia and critical care unit management. However, probably the most important factor for better outcome in cirrhotic patients is based in a careful and accurate patient selection. Preoperative patient selection must be performed by multidisciplinary teams with special focus in hepatic diseases working in referral high-volume center [37].

### Pre-operative management

Although blood tests intended to evaluate preoperative hepatic function in cirrhotic patients requiring liver resection, these techniques frequently underestimate the underlying liver disease [38]. In an attempt to solve this problem, many different methods have been used to evaluate hepatic functional reserve. Among all, the Child-Pugh clinical score is the most validated and used method for cirrhotic patients. Major hepatic resections are safely performed in patients scored as Child- Pugh A but this classification failed to be an adequate predictor of the extent of the liver resection that could be safely performed. In contrast, patients classified as Child- Pugh B and C have unacceptable morbidity and mortality risks and therefore are not suitable for major liver resections [39].

Several methods have been proposed to evaluate hepatic function prior to liver surgery including those based on liver perfusion and biliary excretion (e.g. indocyanine green), on microsomal or cytosolic function (e.g. aminopyrine breath test, galactosyl elimination capacity) and on functional imaging (e.g. 99m-Tc-galactosyl-human serum albumin scintigraphy) [8,40-43]. Redealli et al established in a prospective study that a galactosyl elimination capacity < 4.0 mg/min/Kg is a strong predictor of complications after hepatic surgery in patients with HCC and liver cirrhosis [41]. Among all, the most commonly used is the indocyanine green (ICG) clearance test widely used in eastern countries for cirrhotic patients with hepatocellular carcinoma requiring liver surgery [40,44]. The ICG clearance test evaluated the retention rate of the substance after 15 minutes wich is actively transfer into the hepatic parenchyma, which seems to be the only mechanism involved in metabolism. Finally it is secreted into the bile [8]. The ICG test values are determined by blood sampling or as the percent retention determined by pulsed spectrophotometry in a similar fashion to an oxygen saturation monitor [45]. However, the grater limitation of this test is the variation of results associated with changes in hepatic blood flow. While patients classified as Child-Pugh score A with ICG retention rate <14 % are eligible for large resection, those with results between 15% and 20% are only suitable for limited resections [46].

Some authors combined the results of Child-Pugh score, ICG retention rate at 15 minutes and other biochemical results to define the magnitude of resection, or to decide the use of a preoperative portal embolization to achieve a compensatory hypertrophy of the future liver remnant [1,47]. For example, Wo et al sustained that in patients with ICG retention rate >50%, total bilirubin >4 mg/dl or protrombine time <75%, surgery should not be performed [48]. Taking these parameters into account there have been low mortality rates attributed to liver failure and acceptable morbidity [46,49,50].

However, the grater limitation of these tests is the variation of results associated with changes in hepatic blood flow and others entities affecting hepatic function leading some authors to discuss the accuracy of this method [51,52]. For example, Herold et al found unequal results when comparing tests for hepatic metabolism as galactosyl elimination capacity or aminopyrine breath test with ICG test in patients affected with chronic hepatic diseases [42]. Currently, there is no functional test capable to define on its own the degree of functional reserve and the extent of resection. Therefore, a general algorithm used to evaluate the functional liver reserve in patients with chronic hepatitis or cirrhosis in many centers include clinical evaluation in the search of co-morbidities, blood testing including platelet count and coagulation studies, ICG test, hepatic volume measured by imaging and Child-Pugh classification [44,47].

The presence of severe portal hypertension is an accepted contraindication for liver resection [53]. To exclude this situation, the grade of portal hypertension must be evaluated during preoperative work-up with platelet count, measurement of spleen by imaging studies and endoscopy to assess the presence of esophagus varices [44]. In case of esophagic varices grade III, the patient must be endoscopically treated before surgery [54]. Some authors recommended the systematic measurement of portal pressure gradient to select patients more accurately [11,53]. Bruix et al demonstrated that a portal pressure gradient higher than 10 mmHg was associated with increased morbidity and reduced survival [53]. Furthermore in those with associated high bilirubin mortality was increased [11]. As a consequence, a systematic preoperative measurement of portal pressure gradient has been used in many centers to better select cirrhotic patients with liver tumors [11,53]. Others proposed to delay or avoid hepatectomy in virus related cirrhotic patients with high hepatic transaminases to reduce postoperative mortality [47].

Another important factor to take into account when hepatic regeneration is desired is cholestasis. In this specific situation, most authors agree to perform a preoperative biliary percutaneous drainage of the hemi-liver that will remain after surgery [55]. Nevertheless, many variables such as the type and etiology of the underlying liver disease, the extent and location of the lesions, make the experience of the treating team of primary importance to define the extent of the liver resection in each particular patient [6]. Co-morbidities tend to start or enlarge perioperative complications, so it is sensitive to achieve ASA score I-II prior to surgery because it reduces risk [47]. Moreover, there are entities related to cirrhotic status that can be resolved during surgery to prevent further complications. For example, some authors recommended to perform splenectomy during hepatectomy to reduce postoperative medical complications in patients with severe hyperesplenism [56,57].

Evidence from liver transplant suggests that the donor graft volume must represent ≥ 0.8% of the total body weight of the receptor to reduce the risks of acute hepatic failure [58]. However, the regeneration of chronically ill liver is not equal than that observed in normal organs [8,39,59,60], thus surgeons must be extremely cautious when defining the extent of functional remaining parenchyma to avoid postoperative hepatic failure. Makuuchi et al proposes preoperative right portal vein embolization (PPVE) as a useful attempt to increase the volume of the future remaining liver [61]. Azulay et al demonstrated that resecability can be achieve when PPVE is indicated in cirrhotic patients when the estimated future remnant liver parenchyma ≤40% [62]. When the liver does not regenerate after PPVE, most agree that major hepatectomy should be contraindicated to avoid severe postoperative liver failure [63]. Today, most authors recommend to perform PPVE when the remaining liver is ≥ 25–30% of the whole organ in normal liver and ≥ 40% in injured livers [40,47,49,62]. In eastern countries, where ICG test is widely used in cirrhotic patients, PPVE is recommended when the non-tumoral parenchyma to be removed is ≥ 40% of the organ together with ICG test of 10% to 20% [50].

### Post-Operative Complications

A comparative analysis of morbidity and mortality in cirrhotic patients undergoing liver surgery is very limited as a result of the absence of a standard manner to report postoperative complications. Moreover, most of the studies involve cirrhotic as well as other patients with different hepatic disorders such as fibrosis, cholestasis or hepatitis. Finally, there is no description of severity degree of postoperative complications and often the cause of death is not reported (Table 2).

**Table 2:** Reported postoperative outcome of cirrhotic patients undergoing liver resection.

Authors	Year	Number of patients	Cirrhosis (%)	Overall Complication (%)	Liver Failure (%)	30-day Mortality (%)
Midorikawa et al (50)	1999	173	65	67.6	0^&^	0
Fong et al (86)	1999	154	64.9	45	5	4.5
Torzilli et al (49)	1999	107	59.8	26.2	NA	0
Belghiti (5)	2000	253	94.5	NA	NA	9.5
Takano et al (87)	2000	300	90 ^$^	26 *	NA	4
Zhou et al (66)	2001	1000	88.8	NA	0.5	1.5
Poon et al (46)	2002	206	100	34.9	4.4	5.3^#^
Grazi et al (88)	2003	443	69.5	37	NA	5.9 ^#^
The et al (89)	2005	82	100	NA	11	16

NA: Not Available; ^$ ^series including cirrhosis and other hepatic disorders; * Referred to major complication; ^&^Hepatic insufficiency defined as the presence of both prolonged jaundice (total bilirubin level >3 mg/dL) and encephalopathy; ^# ^Hospital mortality defined as any death occurring during the same hospital admission of the hepatic resection

The evolution of hepatic surgery together with the introduction of novel surgical strategies and modern transection techniques has led to a dramatic reduction in mortality rates from 10–32% [7,64,65] to current series displaying mortality rated of <8% (Table 2). Although post-operative hepatic failure has also been reduced to <5% [46,50,66], most of the different surgical teams evaluate failure in their own fashion.

Many liver surgeons advocated to the conventional approach for the surgical treatment of these patients to reduce hemorrhagic complications. However, some authors have suggested that the laparoscopic approach when feasible can reduce complications related to the abdominal wall or ascitis [67-69]. The use of systematic drainage of abdominal cavity after hepatic surgery in cirrhotic liver has no proven benefits [70,71] and there is also an association with infections, abdominal wall complications and prolonged hospital stay [72].

## Protective strategies in injured livers

Various approaches have been proposed to improve the poorer postoperative outcome of patients with steatosis or cirrhosis after liver surgery. A positive correlation between preoperative liver function, operative time, blood transfusion requirement and postoperative morbidity has been demonstrated [48]. In addition, the extent of blood loss by itself is responsible for short and long term complications [73]. Consequently, inflow occlusion by clamping of the portal triad (Pringle maneuver) has been used since the early 20th century to minimize blood loss during transection of the liver parenchyma in cirrhotic [74] and non-cirrhotic patients [75]. A randomized study confirmed the safety of using inflow occlusion during transection even in cirrhotic livers [76]. This strategy is particularly effective in preventing blood loss but only when associated with low central venous pressure. Anterior approach as a surgical tactic for broad lesions that affect right liver and inhibits its mobilization has proved to be also effective to reduce intra-operative bleeding [77]. Low central venous pressure anesthesia, inflow and/or outflow vascular control, novel transection or coagulation devices are intended all to reduce such loses [38,50].

A recent randomized controlled trial showed that liver transection under inflow occlusion with the clamp crushing technique is associated with lower blood loss and reduced requirement for perioperative transfusions, than resection performed with more sophisticated transection devices claimed to enable safe surgery without the need for inflow occlusion [78]. However, the Pringle maneuver induces ischemic injury in the remnant liver, which is directly related to the duration of inflow occlusion and associated with increased morbidity and mortality [79]. The use of total vascular exclusion during hepatic resection, greatly reduces blood loss, and also ulterior liver regeneration is affected by ischemic organ injury suffered during this process [5]. Two protective strategies such as intermittent clamping (IC) and ischemic preconditioning (IP) intended to reduce ischemic injury and bleeding, however most available clinical data referred to non-cirrhotic patients [67,74,80-82].

There is growing evidence that liver steatosis is associated with reduced outcome after hepatic resection [12]. Since in animal models was observed that steatotic livers poorly tolerate warm ischemia during hepatectomy, many surgeons advocated to develop protective strategies against ischemic injury of these livers [83]. However, animal models applied in experimental studies to protective strategies have all biases precluding the extrapolation of results to the clinical situation [15,83]. The development of clinically relevant experimental models is also hindered by the spectrum of patients with different etiologic factors. Different etiologic backgrounds lead to different forms of steatosis combined with a range of pathologic features unique to some etiologic factors. Furthermore, the clinical significance of the type and extent of steatosis is not clear as larger cohort studies applying uniform diagnostic criteria are missing. In two clinical randomized trial comparing IP or IC versus continuous clamping the highly protective effect of both strategies in steatotic livers was clearly stated [84,85]. In a recent study, the protection conferred by IC was fully preserved in steatotic livers, even if >30% of steatosis was present, while this protective effect was weaker in the IP group [81]. These data may imply that IC should be preferred in patients with severe steatosis and expected prolonged ischemia time. However, more research is needed in this field of surgery as the prevalence of steatosis and hepatitis-related liver cirrhosis is dramatically increasing together worldwide.

## Conclusion

Cirrhosis and, more recently, steatosis are recognized as a clinically important feature that influences patient morbidity and mortality after hepatic resection when compared with patients with healthy liver. In the last years, steatosis has become a major concern as its prevalence is closely linked to obesity, an epidemic disease in Western countries. There is an urgent need for reliable noninvasive methods to detect steatosis and related pathologic features preoperatively.

Surgeons should take the presence of injured livers into account when planning the extent and type of hepatic surgery. Preoperative and perioperative interventions should be considered to minimize the additional damage. The future goal should be to use by consensus a proper classification for morbidity and hepatic failure in liver surgery to compare results between reference centers. These centers should have the commitment to report all deaths related to hepatic failure after surgery.

On choosing a standard of care and treatment for patients with chronic hepatic disease there are no fixed strategies. Each team based on current evidence should decide the best algorithm according to each patient to reach similar rates of morbidity and mortality than those of patients with healthy liver. Further randomized trials should focus on the evaluation of novel preoperative strategies, such as short-term intensive medical treatment of hepatic steatosis and preoperative portal vein embolization, to minimize risk in these patients.

## Abbreviations

ASA: American Society of Anesthesiologists; ICG: indocyanine green; PPVE: Preoperative portal vein embolization; IC: intermittent clamping; IP: ischemic preconditioning

## Competing interests

The authors declare that they have no competing interests.

## Authors' contributions

LM participated in the design of the study, and drafted the manuscript, revising it critically and provided the final approval of the version to be published. PC participated in data acquisition and drafted the manuscript. EQ participated in collection of data and drafted the manuscript. All authors read and approved the final manuscript.
